# Supplementary material for: Heparin-based hydrogel scaffolding alters the transcriptomic profile and increases the chemoresistance of MDA-MB-231 triple-negative breast cancer cells
Source: Biomater Sci. 2020 Feb 13;8(10):2786–96. doi: 10.1039/c9bm01481k (PMC7497406; doi:10.1039/c9bm01481k)
Supplement: Supplementary file 2 [file BM-008-C9BM01481K-s002.zip › Supplementary File 4/HP-BhydrogelvControl/Pathways/my_analysis.Gsea.1545201079907/HALLMARK_UV_RESPONSE_DN.html]

Details for gene set HALLMARK\_UV\_RESPONSE\_DN[GSEA]

|  || Dataset | expr.class.cls#HYD\_versus\_CONTROL.class.cls#HYD\_versus\_CONTROL\_repos |
| Phenotype | class.cls#HYD\_versus\_CONTROL\_repos |
| Upregulated in class | HYD |
| GeneSet | HALLMARK\_UV\_RESPONSE\_DN |
| Enrichment Score (ES) | 0.2259294 |
| Normalized Enrichment Score (NES) | 0.8933953 |
| Nominal p-value | 0.68348044 |
| FDR q-value | 0.8594427 |
| FWER p-Value | 1.0 |
Table: GSEA Results Summary

  

Fig 1: Enrichment plot: HALLMARK\_UV\_RESPONSE\_DN      
 Profile of the Running ES Score & Positions of GeneSet Members on the Rank Ordered List

  

| PROBE | DESCRIPTION (from dataset) | GENE SYMBOL | GENE\_TITLE | RANK IN GENE LIST | RANK METRIC SCORE | RUNNING ES | CORE ENRICHMENT || 1 | CACNA1A | na |  |  | 237 | 2.822 | 0.0177 | Yes |
| 2 | COL1A1 | na |  |  | 499 | 2.347 | 0.0292 | Yes |
| 3 | DBP | na |  |  | 529 | 2.309 | 0.0523 | Yes |
| 4 | PTPRM | na |  |  | 595 | 2.223 | 0.0727 | Yes |
| 5 | CDK13 | na |  |  | 955 | 1.907 | 0.0742 | Yes |
| 6 | VAV2 | na |  |  | 1093 | 1.807 | 0.0864 | Yes |
| 7 | PAPD7 | na |  |  | 1263 | 1.707 | 0.0958 | Yes |
| 8 | DUSP1 | na |  |  | 1364 | 1.645 | 0.1081 | Yes |
| 9 | RUNX1 | na |  |  | 1462 | 1.598 | 0.1201 | Yes |
| 10 | PTPN21 | na |  |  | 1479 | 1.590 | 0.1363 | Yes |
| 11 | SMAD7 | na |  |  | 1487 | 1.581 | 0.1528 | Yes |
| 12 | RBPMS | na |  |  | 1521 | 1.566 | 0.1678 | Yes |
| 13 | ABCC1 | na |  |  | 1714 | 1.485 | 0.1736 | Yes |
| 14 | ERBB2 | na |  |  | 1977 | 1.374 | 0.1746 | Yes |
| 15 | MTA1 | na |  |  | 2016 | 1.355 | 0.1871 | Yes |
| 16 | DYRK1A | na |  |  | 2172 | 1.287 | 0.1927 | Yes |
| 17 | COL5A2 | na |  |  | 2317 | 1.233 | 0.1983 | Yes |
| 18 | ARHGEF9 | na |  |  | 2761 | 1.098 | 0.1868 | Yes |
| 19 | MAGI2 | na |  |  | 2766 | 1.096 | 0.1984 | Yes |
| 20 | DLG1 | na |  |  | 2806 | 1.083 | 0.2079 | Yes |
| 21 | SMAD3 | na |  |  | 2896 | 1.064 | 0.2146 | Yes |
| 22 | RXRA | na |  |  | 3265 | 0.972 | 0.2057 | Yes |
| 23 | IRS1 | na |  |  | 3434 | 0.931 | 0.2068 | Yes |
| 24 | NFKB1 | na |  |  | 3668 | 0.878 | 0.2040 | Yes |
| 25 | MYC | na |  |  | 3771 | 0.854 | 0.2078 | Yes |
| 26 | SYNE1 | na |  |  | 4057 | 0.800 | 0.2014 | Yes |
| 27 | SCAF8 | na |  |  | 4085 | 0.793 | 0.2085 | Yes |
| 28 | PRKCE | na |  |  | 4154 | 0.778 | 0.2132 | Yes |
| 29 | FZD2 | na |  |  | 4195 | 0.772 | 0.2194 | Yes |
| 30 | DLC1 | na |  |  | 4227 | 0.765 | 0.2259 | Yes |
| 31 | PRKCA | na |  |  | 4876 | 0.646 | 0.1989 | No |
| 32 | FYN | na |  |  | 5004 | 0.625 | 0.1989 | No |
| 33 | LDLR | na |  |  | 5135 | 0.608 | 0.1986 | No |
| 34 | SYNJ2 | na |  |  | 5406 | 0.569 | 0.1905 | No |
| 35 | CDC42BPA | na |  |  | 5539 | 0.547 | 0.1894 | No |
| 36 | SLC22A18 | na |  |  | 5759 | 0.518 | 0.1835 | No |
| 37 | MAP2K5 | na |  |  | 5782 | 0.516 | 0.1878 | No |
| 38 | CDKN1B | na |  |  | 6043 | 0.481 | 0.1793 | No |
| 39 | MAPK14 | na |  |  | 6223 | 0.455 | 0.1748 | No |
| 40 | IGF1R | na |  |  | 6443 | 0.424 | 0.1679 | No |
| 41 | NFIB | na |  |  | 6516 | 0.413 | 0.1685 | No |
| 42 | PIK3CD | na |  |  | 6565 | 0.407 | 0.1704 | No |
| 43 | BDNF | na |  |  | 6575 | 0.406 | 0.1742 | No |
| 44 | SLC7A1 | na |  |  | 7059 | 0.353 | 0.1527 | No |
| 45 | RND3 | na |  |  | 7062 | 0.352 | 0.1563 | No |
| 46 | MGLL | na |  |  | 7102 | 0.348 | 0.1580 | No |
| 47 | FHL2 | na |  |  | 7132 | 0.343 | 0.1601 | No |
| 48 | ATP5S | na |  |  | 7309 | 0.324 | 0.1544 | No |
| 49 | KCNMA1 | na |  |  | 7403 | 0.315 | 0.1529 | No |
| 50 | TGFBR3 | na |  |  | 7615 | 0.290 | 0.1449 | No |
| 51 | GRK5 | na |  |  | 7778 | 0.275 | 0.1394 | No |
| 52 | ZMIZ1 | na |  |  | 7808 | 0.271 | 0.1407 | No |
| 53 | PRDM2 | na |  |  | 7819 | 0.269 | 0.1431 | No |
| 54 | COL1A2 | na |  |  | 8100 | 0.246 | 0.1310 | No |
| 55 | NOTCH2 | na |  |  | 8394 | 0.208 | 0.1179 | No |
| 56 | WDR37 | na |  |  | 8532 | 0.201 | 0.1129 | No |
| 57 | KALRN | na |  |  | 8543 | 0.200 | 0.1145 | No |
| 58 | ATXN1 | na |  |  | 8586 | 0.195 | 0.1144 | No |
| 59 | ADORA2B | na |  |  | 8611 | 0.191 | 0.1151 | No |
| 60 | NR1D2 | na |  |  | 8799 | 0.166 | 0.1071 | No |
| 61 | ACVR2A | na |  |  | 8850 | 0.159 | 0.1062 | No |
| 62 | F3 | na |  |  | 9090 | 0.142 | 0.0952 | No |
| 63 | SNAI2 | na |  |  | 9111 | 0.139 | 0.0956 | No |
| 64 | BMPR1A | na |  |  | 9127 | 0.137 | 0.0963 | No |
| 65 | PEX14 | na |  |  | 9195 | 0.126 | 0.0941 | No |
| 66 | LAMC1 | na |  |  | 9285 | 0.120 | 0.0907 | No |
| 67 | YTHDC1 | na |  |  | 9706 | 0.076 | 0.0695 | No |
| 68 | PIAS3 | na |  |  | 9725 | 0.072 | 0.0694 | No |
| 69 | PRKAR2B | na |  |  | 9760 | 0.070 | 0.0683 | No |
| 70 | SCHIP1 | na |  |  | 9768 | 0.069 | 0.0687 | No |
| 71 | SIPA1L1 | na |  |  | 9826 | 0.061 | 0.0663 | No |
| 72 | PDGFRB | na |  |  | 9922 | 0.051 | 0.0619 | No |
| 73 | SFMBT1 | na |  |  | 9988 | 0.042 | 0.0589 | No |
| 74 | DDAH1 | na |  |  | 10115 | 0.026 | 0.0526 | No |
| 75 | RGS4 | na |  |  | 10151 | 0.021 | 0.0510 | No |
| 76 | ATRN | na |  |  | 10622 | -0.019 | 0.0266 | No |
| 77 | MIOS | na |  |  | 10692 | -0.029 | 0.0233 | No |
| 78 | INSIG1 | na |  |  | 11030 | -0.071 | 0.0063 | No |
| 79 | NR3C1 | na |  |  | 11080 | -0.075 | 0.0046 | No |
| 80 | NEK7 | na |  |  | 11301 | -0.089 | -0.0060 | No |
| 81 | MT1E | na |  |  | 11922 | -0.166 | -0.0367 | No |
| 82 | LPAR1 | na |  |  | 11939 | -0.168 | -0.0358 | No |
| 83 | CAP2 | na |  |  | 12189 | -0.199 | -0.0467 | No |
| 84 | ATP2B4 | na |  |  | 12320 | -0.216 | -0.0512 | No |
| 85 | ID1 | na |  |  | 13064 | -0.293 | -0.0870 | No |
| 86 | PIK3R3 | na |  |  | 13096 | -0.298 | -0.0855 | No |
| 87 | ICA1 | na |  |  | 13271 | -0.319 | -0.0912 | No |
| 88 | INPP4B | na |  |  | 13343 | -0.328 | -0.0914 | No |
| 89 | BHLHE40 | na |  |  | 13518 | -0.356 | -0.0967 | No |
| 90 | NRP1 | na |  |  | 13560 | -0.362 | -0.0950 | No |
| 91 | CDON | na |  |  | 13700 | -0.386 | -0.0982 | No |
| 92 | ATP2B1 | na |  |  | 14037 | -0.422 | -0.1113 | No |
| 93 | PPARG | na |  |  | 14161 | -0.436 | -0.1131 | No |
| 94 | APBB2 | na |  |  | 14214 | -0.444 | -0.1110 | No |
| 95 | TGFBR2 | na |  |  | 14301 | -0.464 | -0.1106 | No |
| 96 | AGGF1 | na |  |  | 14398 | -0.476 | -0.1105 | No |
| 97 | CITED2 | na |  |  | 14498 | -0.490 | -0.1105 | No |
| 98 | SDC2 | na |  |  | 15052 | -0.571 | -0.1334 | No |
| 99 | TJP1 | na |  |  | 15122 | -0.585 | -0.1308 | No |
| 100 | AKT3 | na |  |  | 15279 | -0.613 | -0.1324 | No |
| 101 | ATRX | na |  |  | 15309 | -0.618 | -0.1273 | No |
| 102 | NIPBL | na |  |  | 15325 | -0.622 | -0.1214 | No |
| 103 | MAP1B | na |  |  | 15447 | -0.641 | -0.1209 | No |
| 104 | LTBP1 | na |  |  | 15772 | -0.697 | -0.1305 | No |
| 105 | GCNT1 | na |  |  | 15821 | -0.708 | -0.1254 | No |
| 106 | ATP2C1 | na |  |  | 16035 | -0.755 | -0.1285 | No |
| 107 | CAV1 | na |  |  | 16368 | -0.825 | -0.1371 | No |
| 108 | ITGB3 | na |  |  | 16433 | -0.839 | -0.1315 | No |
| 109 | PTGFR | na |  |  | 16553 | -0.866 | -0.1284 | No |
| 110 | CELF2 | na |  |  | 16586 | -0.869 | -0.1208 | No |
| 111 | BCKDHB | na |  |  | 16597 | -0.871 | -0.1120 | No |
| 112 | TFPI | na |  |  | 16601 | -0.872 | -0.1029 | No |
| 113 | DAB2 | na |  |  | 16686 | -0.894 | -0.0977 | No |
| 114 | EFEMP1 | na |  |  | 16779 | -0.921 | -0.0927 | No |
| 115 | HAS2 | na |  |  | 16806 | -0.929 | -0.0841 | No |
| 116 | SPOP | na |  |  | 17037 | -0.997 | -0.0855 | No |
| 117 | PTEN | na |  |  | 17107 | -1.016 | -0.0783 | No |
| 118 | RASA2 | na |  |  | 17300 | -1.084 | -0.0768 | No |
| 119 | ADD3 | na |  |  | 17325 | -1.093 | -0.0663 | No |
| 120 | VLDLR | na |  |  | 17499 | -1.153 | -0.0631 | No |
| 121 | MMP16 | na |  |  | 17559 | -1.181 | -0.0535 | No |
| 122 | PDLIM5 | na |  |  | 17595 | -1.194 | -0.0426 | No |
| 123 | ANXA4 | na |  |  | 17877 | -1.320 | -0.0432 | No |
| 124 | SRI | na |  |  | 17999 | -1.383 | -0.0348 | No |
| 125 | MET | na |  |  | 18018 | -1.394 | -0.0208 | No |
| 126 | PHF3 | na |  |  | 18490 | -1.700 | -0.0274 | No |
| 127 | MRPS31 | na |  |  | 18673 | -1.870 | -0.0169 | No |
| 128 | PMP22 | na |  |  | 18769 | -1.973 | -0.0008 | No |
| 129 | GJA1 | na |  |  | 18902 | -2.173 | 0.0155 | No |
Table: GSEA details [plain text format]

  

Fig 2: HALLMARK\_UV\_RESPONSE\_DN      
 Blue-Pink O' Gram in the Space of the Analyzed GeneSet

  

Fig 3: HALLMARK\_UV\_RESPONSE\_DN: Random ES distribution      
 Gene set null distribution of ES for **HALLMARK\_UV\_RESPONSE\_DN**
